# Exploring the links between sleep quality and stroke risk in older adults

**DOI:** 10.1038/s41598-025-26150-6

**Published:** 2025-11-26

**Authors:** Yanru Xiang, Mingyue Xia, Chunhu Zhang, Puwei Chen, Mei Huang, Xingping Dai

**Affiliations:** 1 Xiangya Hospital, Central South University, Jiangxi (National Regional Center for Neurological Diseases) Honggutan District, No. 266 Fenghe North Avenue, Nanchang, 330038 Jiangxi People’s Republic of China; 2 National Clinical Research Center for Geriatric Disorders’ Xiangya Hospital, Central South University, Changsha, 410008 Hunan People’s Republic of China

**Keywords:** Sleep disturbances, Stroke risk, Older adults, Aging population, Health interventions, Diseases, Health care, Neurology, Neuroscience, Risk factors

## Abstract

The aging population faces significant health challenges, with sleep disturbances emerging as a critical yet often overlooked issue. Poor sleep quality is associated with various adverse health outcomes, including higher risks of mental health disorders and cerebrovascular events such as stroke. Understanding the relationships between sleep health, mental health, and stroke risk is essential for developing effective interventions. This study employed a cross-sectional design involving older adults to examine the associations among sleep disturbances, mental health (specifically depressive symptoms), and stroke risk. Participants completed validated questionnaires assessing sleep quality and depressive symptoms. Multivariate logistic regression and structural equation modeling were employed to analyze the data. The analysis revealed that older adults with difficulty falling asleep faced a significantly increased risk of stroke. Additionally, depressive symptoms were found to mediate the relationship between sleep disturbances and stroke risk. These findings highlight the interconnectedness of sleep health and mental health, demonstrating that poor sleep can contribute to higher rates of depression, which in turn increases stroke risk. The results underscore the importance of prioritizing sleep health in clinical practice for older adults. Addressing sleep disturbances is crucial not only for reducing stroke risk but also for enhancing overall well-being. Future research should further investigate the underlying mechanisms, consider sociocultural factors influencing sleep health, and develop targeted interventions to improve both sleep and mental health in this vulnerable population.

## Introduction

The global trend of population aging presents numerous public health challenges, particularly concerning the well-being of older adults^1^. As individuals transition into later stages of life, they often face a variety of health issues that can significantly impact their quality of life^2,3^. Among the myriad concerns affecting older populations, sleep health emerges as a crucial yet often neglected dimension of overall wellness^4^. Sleep disturbances—encompassing difficulties such as trouble falling asleep, frequent awakenings throughout the night, and waking too early in the morning—are prevalent among older adults and can lead to adverse physical and mental health outcomes^5^.

The implications of poor sleep quality are far-reaching, affecting not only individuals’ mood and cognitive function but also their physiological health^6,7^. Research consistently highlights a strong correlation between sleep disturbances and the prevalence of chronic conditions, including depression, anxiety, and cardiovascular diseases^8,9^. For instance, disruptions in sleep can exacerbate existing health conditions and lead to increased morbidity and mortality rates. Specifically, studies have documented an association between sleep disorders and cardiovascular events, including stroke, which remains a leading cause of disability and death among older adults worldwide^10,11^. Given the overlap between sleep problems and known stroke risk factors such as hypertension, diabetes, and sedentary behavior, understanding the relationship between sleep disturbances and stroke risk is critical for developing effective interventions^12–14^.

Examining the multifaceted relationships between sleep health, mental health, and stroke risk is essential for enhancing the well-being of older adults^15^. Individuals experiencing sleep issues often report higher levels of depressive symptoms, which can further complicate their health status^16^. Furthermore, inadequate sleep may impair cognitive functioning, leading to challenges in everyday activities and reduced quality of life^17,18^. Identifying specific sleep disturbance patterns and their implications for mental health and stroke risk can inform the design of tailored interventions that address these interconnected issues effectively^19^.

In addition to health-related factors, social determinants play a vital role in influencing sleep health and overall well-being among older adults^20^. Engagement in social activities and maintaining strong social support networks can positively impact mental health and sleep quality^21,22^. Exploring how such social factors interact with sleep disturbances can enhance our understanding of the holistic experience of aging and inform community-based programs aimed at supporting older adults in maintaining their health.

Importantly, as the prevalence of sleep disorders continues to grow among older adults, there is a pressing need for holistic approaches that incorporate sleep health into the broader context of aging. Addressing sleep issues through early identification and targeted interventions can ameliorate many of the adverse health outcomes associated with poor sleep. Healthcare systems and policymakers must prioritize the integration of sleep health into the continuum of care for older adults, recognizing the significant contributions that quality sleep makes to physical, mental, and emotional well-being.

The findings from analyses utilizing comprehensive databases provide significant insights into the relationships between sleep health and various health outcomes. These insights can inform recommendations for healthcare providers, policymakers, and community organizations committed to enhancing the health and quality of life for older adults. By emphasizing the importance of good sleep hygiene and addressing sleep-related issues in clinical practice, stakeholders can foster healthier aging and improve overall well-being.

Based on the existing literature and the identified research gaps, this study proposes several interrelated hypotheses. We hypothesize that specific sleep disturbances, including difficulty falling asleep, frequent nighttime awakenings, early morning awakening, and lack of feeling refreshed upon waking, are independently associated with increased stroke risk in older adults, even after controlling for demographic and health-related covariates. Furthermore, we propose that this relationship is mediated through two critical pathways: depressive symptoms (measured by CESD scores) and functional ability (ADL scores). Specifically, we hypothesize that poor sleep quality increases depressive symptoms and impairs daily functioning, which in turn elevates stroke risk. Additionally, we expect that different profiles of sleep disturbances, identified through cluster analysis, will demonstrate distinct associations with mental health outcomes and stroke risk, with more severe sleep disturbance patterns correlating with higher stroke risk and greater mental health burden. Finally, among various sleep disturbance indicators, we hypothesize that difficulty falling asleep will emerge as the most significant predictor of stroke risk when assessed using random forest analysis.

## Methodology

### Study design and data source

The analysis is based on data from the Mexican Health and Aging Study (MHAS), a longitudinal study designed to collect comprehensive data on the health, economic status, and social institutions of older adults in Mexico. This study provides a rich dataset suitable for understanding the dynamics of aging and associated health outcomes. The data used in this analysis were collected between 2001 and 2020, involving multiple waves of interviews to track changes in health status over time ^23,24^.

### Participants

The current analysis included a total of 13,711 participants, comprising 13,236 individuals in the control group without a history of stroke and 475 stroke patients. Participants were selected based on their age (50 years and older) and provided informed consent for participation. The sampling strategy was designed to ensure a representative demographic profile reflective of the older adult population in Mexico, accounting for factors such as age, gender, and urban–rural residence.

### Variables and measurements

Various variables were collected through structured interviews and self-reported questionnaires:

Demographic Variables: Age, gender, marital status, educational attainment, and residential area (urban/rural).

Sleep Disturbances: Assessed through responses to questions regarding the:

1. Difficulty falling asleep (frequency)
2. Waking up at night (frequency)
3. Waking up too early (frequency)
4. Feeling refreshed upon waking (frequency)

Mental Health: Evaluated using the Center for Epidemiologic Studies Depression Scale (CESD) to measure depressive symptoms, with higher scores indicating more severe depression^25,26^.

Functional Ability: Measured using Activities of Daily Living (ADL) scores, reflecting the participants’ ability to perform daily tasks independently^27,28^.

Stroke Risk: Identified through self-reported stroke history, confirmed by medical records when available.

Comorbidities: Information on additional health conditions such as hypertension, diabetes, and cardiovascular diseases.

### Statistical analysis

The statistical analysis involved several sophisticated methods to evaluate the relationships between sleep disturbances, mental health, daily function, and stroke risk.

### Multifactorial logistic regression

Multifactorial logistic regression was employed to assess the associations between sleep disturbances and the risk of stroke. This analysis included three models:

Model 1: Explored the simple relationship between each sleep disturbance and the risk of stroke, providing initial insights into direct associations.

Model 2: Adjusted for demographic variables, such as age, gender, education, and urban–rural residence.

Model 3: Further adjusted for additional health-related variables, including BMI, smoking status, alcohol consumption, and comorbidities (hypertension, diabetes, heart disease).

### Structural equation modeling

Structural Equation Modeling was utilized to analyze potential mediating effects among various factors (e.g., ADL, CESD, cognitive issues, and social interaction) on the relationship between sleep disturbances and stroke risk. SEM allows for the simultaneous examination of multiple relationships and the estimation of direct and indirect pathways.

### Random forest analysis

Random forest analysis was conducted to evaluate the importance of various sleep disturbance variables in predicting stroke risk. This ensemble learning method utilizes multiple decision trees to improve prediction accuracy and assess variable importance.

### Cluster analysis

Cluster analysis was performed to group participants based on their sleep disturbance profiles and associated health outcomes. This method helps identify distinct segments within the population that share similar characteristics related to sleep disturbances, enabling tailored intervention strategies.

The clustering approach revealed three distinct groups:

Cluster 1: Exhibited severe sleep challenges, characterized by high scores for difficulty falling asleep and waking up at night, associated with significant mental and physical health issues.

Cluster 2: Represented individuals with milder disturbances, reflecting better overall health status compared to Cluster 1.

Cluster 3: Included individuals with the least sleep disturbances and favorable health outcomes.

This clustering analysis underscores the necessity for targeted health interventions aimed at specific clusters to improve sleep health and decrease stroke risk.

### Software

All statistical analyses were performed using R (version 4.0). R was particularly employed for advanced statistical modeling, including random forest analysis and structural equation modeling for mediation analysis. Graphical representations were created with R to provide visual summaries of results, enhancing the interpretability and communication of statistical findings.

### Ethical considerations

The study strictly adhered to ethical standards governing research involving human participants, in full compliance with relevant guidelines and regulations. Informed consent was obtained from all participants in the MHAS, and the research protocol was approved by the appropriate institutional review boards. Furthermore, the study ensured participant privacy and data confidentiality, aligning with ethical norms for handling sensitive health data.

## Results

### Characteristics of participants

The characteristics of the study participants, categorized by stroke status, are summarized in Table 1. A total of 13,711 participants were included, comprising 13,236 individuals in the control group and 475 stroke patients.

**Table 1:** Characteristics of participants by stroke status.

Characteristics	Control (N = 13,236)	Stroke(N = 475)	Total(N = 13,711)	*P*-value	FDR
Age					
Mean ± SD	62.74 ± 11.11	68.60 ± 10.54	62.94 ± 11.14		
Median[min–max]	62.00[16.00,101.00]	69.00[32.00,100.00]	62.00[16.00,101.00]		
Gender				0.86	1
Female	5670(41.35%)	206(1.50%)	5876(42.86%)		
Male	7566(55.18%)	269(1.96%)	7835(57.14%)		
Years of Education					
Mean ± SD	7.22 ± 5.09	5.16 ± 4.61	7.15 ± 5.09		
Median[min–max]	6.00[0.0e + 0,22.00]	5.00[0.0e + 0,21.00]	6.00[0.0e + 0,22.00]		
Rural or urban residence				0.74	1
Rural	3787(27.62%)	132(0.96%)	3919(28.58%)		
Urban	9449(68.92%)	343(2.50%)	9792(71.42%)		
BMI					
Mean ± SD	27.93 ± 5.16	27.67 ± 4.91	27.92 ± 5.16		
Median[min–max]	27.34[9.85,69.44]	27.51[8.33,47.92]	27.34[8.33,69.44]		
Alcohol				2.50E−07	2.00E−06
None	9382(68.43%)	389(2.84%)	9771(71.26%)		
Yes	3854(28.11%)	86(0.63%)	3940(28.74%)		
Smoking				0.2	0.79
None	8176(59.63%)	279(2.03%)	8455(61.67%)		
Yes	5060(36.90%)	196(1.43%)	5256(38.33%)		
Self rated health					
Mean ± SD	3.59 ± 0.83	4.00 ± 0.75	3.60 ± 0.83		
Median[min–max]	4.00[1.00,5.00]	4.00[1.00,5.00]	4.00[1.00,5.00]		
Hypertension				1.00E−41	1.60E−40
None	6304(45.98%)	76(0.55%)	6380(46.53%)		
Yes	6932(50.56%)	399(2.91%)	7331(53.47%)		
Diabetes				1.40E−10	1.60E−09
None	9717(70.87%)	285(2.08%)	10,002(72.95%)		
Yes	3519(25.67%)	190(1.39%)	3709(27.05%)		
Heart disease				2.70E−40	4.10E−39
None	11,756(85.74%)	326(2.38%)	12,082(88.12%)		
Yes	1480(10.79%)	149(1.09%)	1629(11.88%)		
Fell in the Last 2 Years				1.70E−11	2.20E−10
None	7937(57.89%)	211(1.54%)	8148(59.43%)		
Yes	5299(38.65%)	264(1.93%)	5563(40.57%)		
Fatigue				4.60E−11	5.50E−10
None	10,845(79.10%)	332(2.42%)	11,177(81.52%)		
Yes	2391(17.44%)	143(1.04%)	2534(18.48%)		

The mean age of participants was significantly higher in the stroke group (68.60 ± 10.54 years) compared to the control group (62.74 ± 11.11 years), and the median age also reflected this difference, with stroke patients having a median age of 69.0 years versus 62.0 years in controls. The majority of participants were male, constituting 55.18% of the control group and 56.57% of the stroke group; however, the gender distribution did not show significant differences between groups (*p* = 0.86).

Educational attainment was notably lower among stroke patients, with a mean of 5.16 ± 4.61 years compared to 7.22 ± 5.09 years in the control group. The data revealed a predominance of urban residents in both groups, with no significant differences (*p* = 0.74). BMI was similar across groups, with no statistically significant difference observed.

Alcohol consumption was significantly associated with stroke status, indicating more participants in the control group reported no alcohol use compared to the stroke group (*p* = 2.50E−07). In contrast, smoking status did not reveal significant differentiation (*p* = 0.20).

Health-related variables showed considerable disparities; stroke patients reported lower self-rated health scores (mean 4.00 ± 0.75) compared to controls (mean 3.59 ± 0.83). Hypertension and diabetes prevalence were significantly higher in stroke patients, with *p*-values of 1.00E−41 and 1.40E−10, respectively. Heart disease was also more prevalent among stroke patients (*p* = 2.70E−40). Furthermore, fatigue was observed in a higher percentage of stroke survivors compared to controls (*p* = 4.60E−11). These results highlight significant differences in demographic and health behaviors between stroke patients and controls.

The characteristics of participants stratified by stroke status, highlighting the prevalence of sleep disturbances and activity levels (Table 2). A total of 13,711 participants were analyzed, with 13,236 in the control group and 475 in the stroke cohort.

**Table 2:** Characteristics of participants by sleep disturbance and activity level.

Characteristics	Control(N = 13,236)	Stroke(N = 475)	Total(N = 13,711)	p-value	FDR
Difficulty falling asleep frequency				7.70E−08	7.70E−07
None	11,597(84.58%)	376(2.74%)	11,973(87.32%)		
Yes	1639(11.95%)	99(0.72%)	1738(12.68%)		
Frequency of waking up at night				1.00E−06	7.30E−06
None	11,084(80.84%)	357(2.60%)	11,441(83.44%)		
Yes	2152(15.70%)	118(0.86%)	2270(16.56%)		
Frequency of waking up too early				3.80E−04	1.90E−03
None	10,593(77.26%)	348(2.54%)	10,941(79.80%)		
Yes	2643(19.28%)	127(0.93%)	2770(20.20%)		
Frequency of feeling refreshed upon waking				8.70E−05	5.20E−04
None	5694(41.53%)	248(1.81%)	5942(43.34%)		
Yes	7542(55.01%)	227(1.66%)	7769(56.66%)		
ADL					
Mean ± SD	0.29 ± 0.88	1.00 ± 1.61	0.31 ± 0.93		
Median[min–max]	0.0e + 0[0.0e + 0,6.00]	0.0e + 0[0.0e + 0,6.00]	0.0e + 0[0.0e + 0,6.00]		
CESD					
Mean ± SD	3.16 ± 2.57	4.35 ± 2.83	3.20 ± 2.58		
Median[min–max]	3.00[0.0e + 0,9.00]	4.00[0.0e + 0,9.00]	3.00[0.0e + 0,9.00]		
Memory impairment				2.10E−13	2.90E−12
Fair	868(6.33%)	21(0.15%)	889(6.48%)		
Good	4597(33.53%)	107(0.78%)	4704(34.31%)		
Poor	550(4.01%)	14(0.10%)	564(4.11%)		
Very good	6486(47.31%)	276(2.01%)	6762(49.32%)		
Excellent	735(5.36%)	57(0.42%)	792(5.78%)		
Cognitive problems				1	1
None	13,192(96.21%)	473(3.45%)	13,665(99.66%)		
Yes	44(0.32%)	2(0.01%)	46(0.34%)		
Frequency of participation in social activities					
Mean ± SD	4.39 ± 3.49	3.55 ± 3.40	4.36 ± 3.49		
Median[min–max]	3.00[1.00,9.00]	1.00[1.00,9.00]	3.00[1.00,9.00]		
Frequency of contact with family and friends					
Mean ± SD	6.41 ± 3.00	5.87 ± 3.19	6.39 ± 3.01		
Median[min–max]	7.00[1.00,9.00]	7.00[1.00,9.00]	7.00[1.00,9.00]		
Activity				2.00E−07	1.80E−06
None	8450(61.63%)	359(2.62%)	8809(64.25%)		
Yes	4786(34.91%)	116(0.85%)	4902(35.75%)		

Significant differences were observed in sleep disturbances between the two groups. The prevalence of Difficulty Falling Asleep was 11.95% in the control group compared to 21.05% in the stroke group (*p* = 7.70E–08). The Frequency of Waking Up at Night was reported by 15.70% of controls and 24.84% of stroke patients (*p* = 1.00E−06). Additionally, Waking Up Too Early was noted in 19.28% of the control group versus 26.68% of stroke group participants (*p* = 3.80E−04). Conversely, a lower percentage of stroke patients (55.01%) reported feeling refreshed upon waking compared to 41.53% of control participants (*p* = 8.70E−05).

In terms of ADL, stroke patients exhibited a significantly higher mean score (1.00 ± 1.61) than that of controls (0.29 ± 0.88), although both groups showed a median of 0.0. The CESD scores reflected a higher mean in stroke patients (4.35 ± 2.83) than in controls (3.16 ± 2.57), indicating a statistically significant difference (*p* < 0.001).

Memory impairment was markedly more prevalent among stroke participants, characterized by a mere 0.15% reporting their memory as fair, in contrast to 6.33% in the control group (*p* = 2.10E−13). Cognitive problems were infrequent, with 96.21% of the control group reporting none, compared to 96.9% in the stroke group (*p* = 1).

The frequency of participation in social activities was significantly lower in stroke patients, averaging 3.55 ± 3.40 compared to 4.39 ± 3.49 in controls. Similarly, contact with family and friends also differed, with stroke patients recording a mean of 5.87 ± 3.19 versus 6.41 ± 3.00 among controls (*p* = 2.00E−07). Moreover, 2.62% of stroke patients experienced limitations in physical activity compared to 61.63% in the control group, indicating a substantial impact of stroke on social engagement and daily functioning.

### Random forest analysis on sleep disturbances

The importance scores and standard deviations for various sleep disturbances as assessed by random forest analysis (Table 3). The analysis highlights the contribution of each variable to the predictive model concerning stroke risk.

**Table 3:** Importance scores of sleep disturbances in random forest analysis.

Variable	Importance score	Standard deviation
Difficulty falling asleep frequency	27.06	13.24
Frequency of waking up too early	26.18	14.75
Frequency of waking up at night	25.38	13.69
Frequency of feeling refreshed upon waking	24.05	16.57

The variable Difficulty Falling Asleep Frequency emerged as the most significant predictor, with an importance score of 27.06 and a standard deviation of 13.24. This indicates a strong influence on the outcome of interest, underscoring the relevance of sleep initiation problems in relation to stroke risk.

Following closely, the Frequency of Waking Up Too Early was assigned an importance score of 26.18, with a standard deviation of 14.75. This finding suggests that experiencing early awakenings is also a critical feature linked to the evaluated health outcomes.

The Frequency of Waking Up at Night ranked third, with an importance score of 25.38 and a standard deviation of 13.69. This result indicates that interruptions during the night are pertinent factors that contribute to the predictive accuracy of stroke assessments.

Lastly, the Frequency of Feeling Refreshed Upon Waking received the lowest importance score of 24.05, alongside a higher standard deviation of 16.57. While still significant, this variable appears to have a slightly lesser impact on the stroke risk evaluation compared to the others.

### Multinomial logistic regression for sleep disturbances and stroke

The multivariate logistic regression analysis evaluated the relationship between sleep disturbances and stroke risk through three distinct models for each variable. This detailed assessment provided insights into how specific sleep-related issues correlate with stroke incidence.

**Fig. 1:**
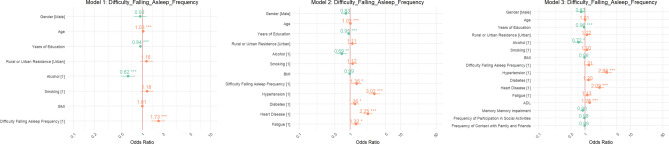
Odds ratios for frequency of Difficulty Falling Asleep Frequency.

For Difficulty Falling Asleep Frequency (Fig. 1), the OR in Model 1 was 2.60 (95% CI 1.45–4.65), indicating that individuals reporting difficulty falling asleep were 2.6 times more likely to experience a stroke compared to those without this issue. In Model 2, the OR rose to 3.10 (95% CI 1.72–5.54), further emphasizing the strong link between this disturbance and stroke risk. Ultimately, in Model 3, the OR reached 3.00 (95% CI 1.67–5.34), reinforcing the conclusion that people struggling to fall asleep face more than triple the odds of stroke occurrence.

**Fig. 2:**
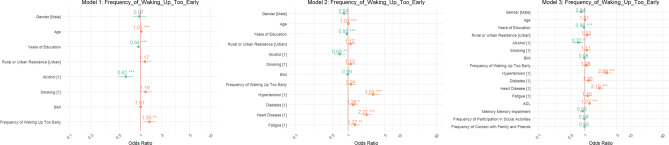
Odds ratios for Frequency of Waking Up Too Early.

Regarding the Frequency of Waking Up Too Early (Fig. 2), Model 1 presented an OR of 1.90 (95% CI 1.12–3.22), suggesting a substantial increase in stroke risk for individuals waking up too early. This association became more pronounced in Model 2, with the OR climbing to 2.00 (95% CI 1.18–3.38). By Model 3, the OR peaked at 2.20 (95% CI 1.32–3.68), demonstrating that this sleep disturbance significantly contributes to heightened stroke risk.

**Fig. 3:**
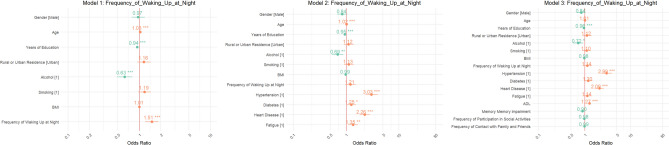
Odds ratios for frequency of waking up at night.

The analysis of the Frequency of Waking Up at Night (Fig. 3) revealed notable associations with stroke risk as well. In Model 1, the odds ratio was calculated at 1.93 (95% CI 1.12–3.34), indicating individuals who frequently wake at night had nearly double the odds of suffering a stroke. This risk escalated significantly in Model 2, yielding an OR of 3.00 (95% CI 1.75–5.15). By Model 3, the OR remained at 3.00, underscoring a persistent and critical association between nighttime awakenings and increased stroke risk.

**Fig. 4:**
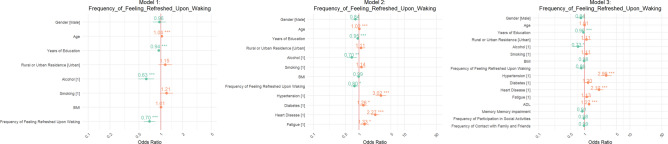
Odds ratios for Frequency of Feeling Refreshed Upon Waking.

In contrast, the Frequency of Feeling Refreshed Upon Waking (Fig. 4) demonstrated a protective effect against stroke. In Model 1, the OR stood at 0.58 (95% CI 0.36–0.92), indicating a 42% reduction in the odds of stroke for those feeling refreshed. This protective association was supported by Model 2, where the OR was 0.54 (95% CI 0.34–0.84). Ultimately, in Model 3, the odds ratio further decreased to 0.40 (95% CI 0.23–0.71), confirming that individuals who wake up feeling refreshed are significantly less likely to experience a stroke.

### Mediation analysis results

The mediation analysis evaluated the relationships among sleep disturbances, ADL, CESD, memory impairment, cognitive issues, and social interaction. The estimates, standard errors, and significance levels provide insight into these associations (Table 4).

**Table 4:** Estimates of sleep disturbances on health measures: a mediation analysis.

Dependent Variable	Independent variable	Estimate	Standard Error	z-value	*p*-Value
ADL	Difficulty falling asleep frequency	0.309	0.026	11.73	< 0.001
ADL	Frequency of waking up at night	0.164	0.024	6.89	< 0.001
ADL	Frequency of waking up too early	0.089	0.021	4.14	< 0.001
ADL	Frequency of feeling refreshed upon waking	− 0.135	0.016	− 8.53	< 0.001
CESD	Difficulty falling asleep frequency	1.325	0.068	19.44	< 0.001
CESD	Frequency of waking up at night	0.647	0.061	10.52	< 0.001
CESD	Frequency of waking up too early	0.714	0.055	12.89	< 0.001
CESD	Frequency of feeling refreshed upon waking	− 1.334	0.041	− 32.47	< 0.001
Memory Impairment	Difficulty falling asleep frequency	− 0.154	0.025	− 6.25	< 0.001
Memory impairment	Frequency of waking up at night	− 0.133	0.022	− 6	< 0.001
Memory impairment	Frequency of waking up too early	− 0.09	0.02	− 4.49	< 0.001
Memory impairment	Frequency of feeling refreshed upon waking	0.148	0.015	9.97	< 0.001
Cognitive problems	Difficulty falling asleep frequency	0.003	0.002	1.81	0.071
Cognitive problems	Frequency of waking up at night	0.001	0.002	0.88	0.377
Cognitive problems	Frequency of waking up too early	0.00002	0.001	0.01	0.989
Cognitive problems	Frequency of feeling refreshed upon waking	0.001	0.001	0.99	0.324
Frequency of participation in social activities	Difficulty falling asleep frequency	0.204	0.101	2.02	0.044
Frequency of participation in social activities	Frequency of waking up at night	− 0.049	0.091	− 0.54	0.588
Frequency of participation in social activities	Frequency of waking up too early	0.233	0.082	2.84	< 0.001
Frequency of participation in social activities	Frequency of feeling refreshed upon waking	0.097	0.061	1.59	0.113
Frequency of contact with family and friends	Difficulty falling asleep frequency	0.235	0.087	2.69	0.007
Frequency of contact with family and friends	Frequency of waking up at night	0.149	0.079	1.9	0.058
Frequency of contact with family and friends	Frequency of waking up too early	− 0.072	0.071	− 1.02	0.308
Frequency of contact with family and friends	Frequency of feeling refreshed upon waking	0.407	0.052	7.75	< 0.001
Stroke	ADL	0.024	0.002	14.4	< 0.001
Stroke	CESD	0.03	0.001	5.43	< 0.001
Stroke	Memory impairment	− 0.06	0.002	− 3.14	0.002
Stroke	Cognitive problems	− 0.017	0.027	− 0.64	0.522
Stroke	Frequency of participation in social activities	− 0.012	0	− 3.7	< 0.001
Stroke	Frequency of contact with family and friends	− 0.021	0.001	− 1.66	0.096

For ADL, difficulty falling asleep frequency had a strong positive association (estimate = 0.309), indicating that increased difficulty in falling asleep correlates with reduced functionality. Waking up at night also showed a substantial positive impact (estimate = 0.164), while waking up too early was similarly linked (estimate = 0.089). Conversely, feeling refreshed upon waking had a negative estimate (–0.135), suggesting poor sleep quality negatively affects daily activities.

For CESD, difficulty falling asleep was significantly related (estimate = 1.325), indicating a strong link between sleep issues and increased depressive symptoms. Waking up at night (estimate = 0.647) and waking up too early (estimate = 0.714) further confirmed this relationship. Notably, feeling refreshed upon waking had a substantial negative estimate of – 1.334, emphasizing its protective effect against depression.

In terms of memory impairment, all sleep disturbances exhibited negative associations. Difficulty falling asleep had an estimate of – 0.154, indicating a link to decreased performance. Waking up at night yielded – 0.133, while waking up too early also showed a negative association ( − 0.090). Feeling refreshed upon waking positively correlated with memory (estimate = 0.148).

For cognitive problems, no significant associations were evident.

Regarding social activities, difficulty falling asleep was positively associated (estimate = 0.204), and waking up too early also had a significant effect (estimate = 0.233). Waking up at night had a non-significant estimate ( − 0.049), indicating it did not significantly impact social participation.

In the context of contact with family and friends, difficulty falling asleep positively related (estimate = 0.235), and feeling refreshed had a strong positive estimate of 0.407. Waking up at night was marginally non-significant (estimate = 0.149).

Lastly, in assessing stroke, ADL (estimate = 0.024) and CESD (estimate = 0.030) showed significant positive relationships, while memory impairment had a negative association (estimate = − 0.060). Cognitive problems had no significant relationship (estimate = − 0.017).

### Sleep disturbance clusters: implications for mental health and stroke

The data presents three clusters of individuals based on sleep disturbance characteristics and associated outcomes, including fatigue, CESD, and stroke risk (Table 5). Cluster 1 exhibits significant sleep challenges, characterized by high scores for difficulty falling asleep (1.4431) and waking up at night (1.4401). Individuals also frequently wake up too early (1.2241) and report feeling unrefreshed upon waking (– 0.4537), suggesting poor sleep quality. This correlates with high daytime fatigue (0.7531) and considerable depressive symptoms (0.8514), alongside a substantial stroke risk score of 5.2786, indicating severe health concerns. In contrast, Cluster 2 shows relatively minor sleep issues, with lower scores for difficulty falling asleep (0.2454), waking up at night (0.2229), and waking up too early (0.1627). While these individuals experience moderate fatigue (0.2995) and some lack of refreshment (– 0.1791), they have lower depressive symptoms (0.4444) and minimal stroke risk (– 0.1734), suggesting a healthier profile. Lastly, Cluster 3 presents the most favorable outcomes, with lower scores for sleep disturbances and low fatigue levels (– 0.1731). Individuals feel somewhat refreshed upon waking (0.1042) and have decreased depressive symptoms (–0.2003), with a stroke risk score of –0.1894, further highlighting their overall better health profile. This analysis illustrates a strong relationship between sleep disturbances and mental health outcomes, underscoring the need for targeted interventions to improve sleep health and mitigate associated risks.

**Table 5:** Health outcomes associated with sleep disturbance clusters.

Cluster	Difficulty falling asleep frequency	Frequency of waking up at night	Frequency of waking up too early	Frequency of feeling refreshed upon waking	Fatigue	CESD	Stroke
1	1.4431	1.4401	1.2241	− 0.4537	0.7531	0.8514	5.2786
2	0.2454	0.2229	0.1627	− 0.1791	0.2995	0.4444	− 0.1734
3	− 0.3174	− 0.3158	− 0.2672	0.1042	− 0.1731	− 0.2003	− 0.1894

## Discussion

The findings of this study provide significant insights into the complex relationships between sleep disturbances, mental health, and the risk of stroke among older adults. As this population segment continues to grow, understanding these associations becomes increasingly vital for healthcare providers and policymakers.

### Overview of key findings

This study identified significant relationships between sleep disturbances and health outcomes. Most notably, difficulty falling asleep was strongly associated with increased risk of stroke. Participants with significant sleep disturbances also exhibited higher rates of depressive symptoms, as measured by the CESD. These findings align with existing research highlighting the detrimental effects of poor sleep on both mental health and overall well-being.

The multifactorial logistic regression results indicated that older adults experiencing chronic sleep disturbances face a markedly elevated likelihood of stroke. Specifically, difficulty falling asleep emerged as a crucial predictor of stroke risk, underscoring the need for healthcare providers to prioritize sleep evaluations in these patients. Additionally, SEM revealed that depressive symptoms mediate the relationship between sleep disturbances and stroke risk, suggesting that improvements in sleep quality could alleviate depression and reduce stroke risk among older adults.

### Implications for clinical practice

Given the significant associations identified in this study, several key implications for clinical practice emerge. First and foremost, there is a pressing need to integrate sleep health assessments into routine healthcare evaluations for older adults^29,30^. Healthcare providers should routinely screen for sleep disturbances, particularly in patients at higher risk of stroke or those who report mental health issues. Utilizing standardized screening tools, such as the Pittsburgh Sleep Quality Index (PSQI), can facilitate early identification of sleep problems and guide subsequent interventions.

Moreover, interdisciplinary approaches to managing sleep disturbances are essential. Collaborating with specialists in sleep medicine, psychiatry, and gerontology can enhance the quality of care provided to older adults. For example, interventions such as cognitive-behavioral therapy for insomnia (CBT-I) can be effective in treating sleep disturbances while also addressing co-morbid depression. By taking a holistic approach that considers both mental and physical health, healthcare providers can implement comprehensive treatment plans tailored to the individual needs of older adults.

Education and awareness-raising among both patients and healthcare providers are crucial components in improving sleep health. Many older adults may not recognize the significance of their sleep issues or may attribute insomnia to the aging process. Therefore, outreach programs that emphasize the importance of sleep, provide practical strategies for improving sleep hygiene, and challenge ageist perceptions surrounding sleep can empower older adults to take an active role in their health management.

### Limitations

While the findings from this study offer valuable insights, certain limitations warrant consideration. First, the cross-sectional nature of the analysis limits the ability to draw causal inferences regarding sleep disturbances, mental health, and stroke risk. Future longitudinal studies are needed to establish temporal relationships and clarify the directionality of these associations. Such studies could also explore how interventions targeting sleep disturbances might affect mental health and stroke risk over time.

Second, self-reported measures of sleep disturbances and mental health may be subject to biases, including social desirability and recall bias. Incorporating objective measures of sleep, such as actigraphy or polysomnography, could enhance the validity of findings and provide a comprehensive understanding of sleep patterns among older adults.

This study makes important contributions to clinical practice and stroke prevention strategies. Our findings provide evidence-based support for integrating routine sleep assessment into stroke prevention programs, particularly in primary care settings, with specific sleep disturbances (difficulty falling asleep, nighttime awakenings, early morning awakening) serving as clear screening targets. The demonstrated mediating role of depressive symptoms suggests that interventions targeting both sleep quality and mental health could yield synergistic benefits in reducing stroke incidence while promoting overall health and quality of life. Additionally, the identified sleep disturbance clusters enable clinicians to develop personalized intervention strategies based on individual risk profiles, emphasizing the importance of multidisciplinary care coordination among sleep specialists, neurologists, and primary care physicians. Future research should prioritize several critical directions: prospective longitudinal studies to establish temporal causality and evaluate whether sleep interventions can reduce stroke incidence; incorporation of objective sleep measurement methods such as polysomnography to complement self-reported data; investigation of biological mechanisms including inflammatory biomarkers and autonomic dysfunction; randomized controlled trials testing sleep interventions for stroke prevention; cross-cultural studies examining sociocultural influences on the sleep-stroke relationship; and research exploring interactions between sleep disturbances and other modifiable stroke risk factors to identify optimal combination intervention strategies for comprehensive stroke prevention in aging populations.

## Conclusion

This study provides significant evidence of the relationships between sleep disturbances, mental health, and stroke risk among older adults. The findings emphasize the need to prioritize sleep health in clinical practice and adopt comprehensive, interdisciplinary care approaches. As the aging population grows, addressing sleep issues is essential for reducing stroke risk and enhancing overall health and quality of life. Future research should investigate the mechanisms behind these relationships, consider sociocultural factors affecting sleep health, and develop effective interventions targeting both sleep and mental health. Such efforts will promote healthier aging and improve outcomes for older individuals.

## Data Availability

The data used in the present study are all publicly available at https://mhasweb.org/Home/index.aspx.
